# Targeting oxidative stress to reduce osteoarthritis

**DOI:** 10.1186/s13075-015-0908-7

**Published:** 2016-01-27

**Authors:** Blandine Poulet, Frank Beier

**Affiliations:** Institute of Ageing and Chronic Disease, University of Liverpool, Liverpool, UK; Department of Physiology and Pharmacology, Schulich School of Medicine & Dentistry, University of Western Ontario, Children’s Health Research Institute, London, Ontario Canada

## Abstract

Osteoarthritis (OA) is the commonest chronic disease, with an estimated 9.6 % of men and 18.0 % of women aged over 60 years having symptomatic OA according to the World Health Organisation. Despite this prevalence, no therapies to slow disease progression are currently available. Oxidative stress has been described as an important factor in various diseases, and more recently in OA. Evidence for using antioxidants to reduce OA severity is slowly accumulating but further understanding of their chondroprotective mechanisms in joint tissues is still required to demonstrate potential benefit to patients. A new study implicates the transcriptional repressor Bach-1 and its downstream target HO-1 as important players in this process.

## Editorial

Osteoarthritis (OA) is a complex multifactorial degenerative disease of the joint, with risk factors including ageing, mechanical disturbance, genetics and obesity. Despite a high prevalence, no therapies to prevent or slow disease progression are currently available. The hallmarks of OA involve degradation of the extracellular matrix of the articular cartilage, subchondral bone sclerosis, synovial membrane activation and thickening, and osteophyte formation. Various pathways have also been linked to OA, in particular those involved in articular chondrocyte phenotypic changes.

In a recent article of *Arthritis Research & Therapy*, a novel link between OA and the regulation of oxidative stress in chondrocytes has been proposed, with potential new targets for therapy [1]. Oxidative stress is known to be detrimental to many cells and to occur during disease and with ageing. It has also been implicated in the development of OA (reviewed in [2]). Thus, research on the effects of antioxidants on OA pathogenesis is being performed by various groups.

In the study by Takada et al. [1], two distinct preclinical mouse models of OA were used, ageing and surgical mechanical instability of the knee joint, in a mouse deficient in BTB and CNC homology 1 (Bach 1). Bach 1 is a negative transcriptional regulator of the antioxidant Heme oxygenase-1 (HO-1). The deficiency in Bach-1 promoted HO-1 expression in mice, and protected murine knee joints from OA development in primary ageing and post-traumatic models of disease. Bach-1 deficiency in vitro also led to decreased matrix metalloproteinase (MMP)-13 and ADAMTS-5 (A disintegrin and metalloproteinase with thrombospondin motifs 5) gene expression in response to cytokine treatment, changes concurrent with a protection against cartilage loss. This protection was thought to be achieved via increased autophagy, essential for cellular homeostasis, and decreased apoptosis. At least some of these protective effects of Bach-1 deficiency were lost when chondrocytes were treated with small interfering RNAs against HO-1. From these data, the authors conclude that the maintenance of HO-1 levels, via inhibition of Bach-1, may be used to protect against OA development in both ageing and post-traumatic OA.

This study complements recent findings from other groups. Another regulator of antioxidants such as HO-1 is the transcription factor nuclear factor (erythroid-derived 2)-like 2 (Nrf2). Deletion of Nrf2 resulted in increased OA severity in a murine post-traumatic model of OA (destabilisation of the medial meniscus (DMM)) and in the inflammatory model induced by monosodium iodoacetate injection [3]. In addition, in that study, Cai et al. used a histone deacetylase inhibitor (trichostatin A) to induce Nrf2 activation and HO-1 expression, and to repress interleukin (IL)-1β-induced MMP gene expression in chondrocytes. Similarly, trichostatin A treatment in vivo resulted in decreased OA severity and reduced MMP expression in mice, as well as increased HO-1. These effects were shown to be dependent on the presence of Nrf2.

Sulfurophane is a natural potent activator of Nrf2 [4]. Sulfurophane was shown to induce HO-1 gene expression, as a downstream target of Nrf2. In that study, Davidson et al. also showed that treatment reduced IL-1β-induced MMP gene expression, but independently of Nrf2. Sulfurophane was also able to reduce cytokine-induced cartilage degradation in vitro and cartilage degradation in the DMM model in vivo. These studies, in conjunction with the Takada et al. paper, provide strong evidence that upregulation of HO-1 is a promising strategy to protect from inflammatory and post-traumatic OA. However, the role of HO-1 itself still has to be examined in models of OA.

Takada et al. also showed that Bach-1 deficiency increased autophagy, which could be linked to the OA protection seen in these mice. Indeed, autophagy is a protective mechanism of cell survival in response to stress. Activation of autophagy in post-traumatic OA, using the mTOR (mechanistic target of rapamycin) inhibitor rapamycin, leads to decreased OA severity, with concurrent reductions in OA markers MMP-13 and collagen X and increases in the autophagy marker LC3 (microtubule-associated protein 1 light chain 3 alpha) [5]. Similarly, deletion of mTOR from chondrocytes, a strong repressor of autophagy, leads to protection from OA development with significant reduction of cartilage degradation, apoptosis and synovial fibrosis [6].

It seems from these studies that maintaining high levels of HO-1 is indeed beneficial against OA development, although the mechanisms involved in the chondroprotective effects of antioxidants are complex and act on various pathways, including Nrf2-controlled antioxidant response elements and autophagy. A further understanding of these pathways and their regulation will lead to important novel targets to slow OA progression.

## Abbreviations

DMM: destabilisation of the medial meniscus
IL: interleukin
MMP: matrix metalloproteinase
mTOR: mechanistic target of rapamycin
OA: osteoarthritis
